# Extracellular Vesicles in B-Cell Non-Hodgkin Lymphomas: Pathogenesis, Therapeutic Implications, and Biomarker Potential

**DOI:** 10.3390/biomedicines14040767

**Published:** 2026-03-27

**Authors:** Tingjun Zhu, Jingcheng Zhang

**Affiliations:** Affiliated Jinhua Hospital, Zhejiang University School of Medicine, Jinhua 321000, China; zhutingjunztj@163.com

**Keywords:** B-cell non-Hodgkin lymphomas, extracellular vesicles, small EVs, drug resistance, biomarkers, tumor microenvironment

## Abstract

Extracellular vesicles (EVs), as key mediators of intercellular communication, play multifaceted roles in the pathogenesis, treatment, drug resistance, and monitoring of B-cell non-Hodgkin lymphomas (B-NHLs), including diffuse large B-cell lymphoma (DLBCL), Burkitt lymphoma (BL), follicular lymphoma (FL), and mantle cell lymphoma (MCL). EVs derived from lymphoma cells or tumor microenvironment cells carry diverse cargoes such as proteins, microRNAs (miRNAs), and viral oncoproteins, which regulate tumor progression by modulating signaling pathways related to cell proliferation, invasion, apoptosis, autophagy, and immune suppression. In terms of treatment, accumulating evidence suggests that EVs may be associated with the efficacy of classical regimens such as R-CHOP, and they also hold potential as therapeutic targets and drug delivery vehicles for B-NHL. They contribute to drug resistance by altering the expression of key molecules or reshaping the tumor niche. Additionally, EV-derived biomarkers enable non-invasive diagnosis and monitoring of treatment response and prognosis. This review summarizes the latest research progress on the roles of EVs in major B-NHL subtypes, aiming to provide new insights for the development of innovative diagnostic and therapeutic strategies for B-NHL.

## 1. Introduction

The incidence rate of B-cell lymphoma in Western countries stands at 20 per 100,000 [1]. This disease can affect any organ, leading to a wide array of distinct clinical manifestations. The prognosis varies significantly among different types of B-cell lymphomas, and there is also considerable heterogeneity within the same type of lymphoma. The pathophysiological mechanisms underlying this disease have not yet been fully elucidated. Current treatment strategies for B-cell lymphoma predominantly rely on CD20 monoclonal antibodies (CD20 mAb) in combination with conventional chemotherapy. While the majority of patients achieve long-term survival, a subset of individuals exhibits suboptimal treatment response or early disease recurrence. This is particularly true for patients with high-risk factors, who consistently demonstrate poor clinical outcomes.

EVs are lipid bilayer-enclosed subcellular vesicles with an average diameter of approximately 100 nm. They are ubiquitously present in bodily fluids, where they carry and deliver signaling molecules to modulate cellular physiological states and are closely linked to the initiation and progression of various diseases. In 1981, E.G. Trams first identified bilayer membrane-bound small vesicles in the supernatant of in vitro-cultured sheep reticulocytes, which were later named “exosomes” by Johnstone in 1987. In accordance with the MISEV2018 guidelines, we herein use the term “small EVs” (small extracellular vesicles) to refer to a heterogeneous population of lipid-bilayer-enclosed particles released by cells, regardless of their biogenesis, and distinguishable from larger microvesicles by their size and physical properties [2]. Once considered cellular waste-disposal vesicles, EVs have since been increasingly recognized as key mediators of intercellular communication with advancing research. They participate in diverse biological processes, including antigen presentation, cell differentiation, cell growth, tumor immune responses, and tumor cell migration. Notably, EVs also exert a pivotal influence on the occurrence, development, and chemoresistance of B-cell lymphoma.

**Figure 1:**
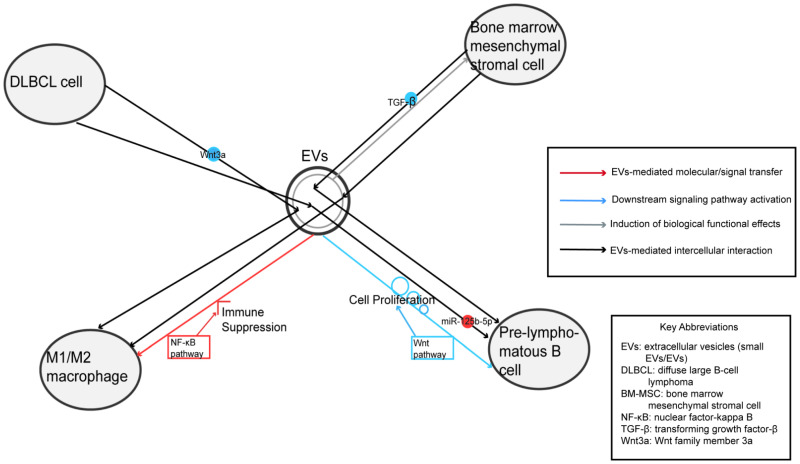
Overview of EVs mediated intercellular communication and pathogenic mechanisms in B-NHL.

While systemic metabolic conditions (e.g., obesity, diabetes) are risk factors for lymphoma, and cellular metabolism is reprogrammed in cancer, direct evidence linking EV-mediated systemic metabolic dysregulation to B-NHL progression remains limited in the current literature. Most documented EV effects in B-NHL are immunomodulatory or involve direct signal transduction (e.g., NF-κB, Wnt) rather than explicit metabolic reprogramming (e.g., glycolysis, lipid synthesis) transferred via EVs. This article primarily elaborates on the intricate interplay between lymphoma and EVs, focusing on key aspects including pathogenesis, therapeutic applications, drug resistance mechanisms, and clinical monitoring (Figure 1).

## 2. Diffuse Large B-Cell Lymphoma (DLBCL)

DLBCL is the most prevalent subtype of NHL, characterized by strong invasiveness and marked biological heterogeneity. A subset of DLBCL patients further faces challenges of chemotherapy resistance and disease recurrence, posing significant hurdles to clinical management. EV-specific biological functions in DLBCL remain poorly defined.

### 2.1. EV-Mediated Pathogenesis

EVs originating from DLBCL cells transport biological molecules that foster tumor progression and survival. Specifically, these molecules enhance cell proliferation and invasive capacity, boost chemoresistance, and dampen apoptotic processes [3,4,5,6,7,8,9]. According to the findings from Koch R et al., [10] DLBCLs are composed of clonogenic side population (SP) cells and non-SP cells organized in a dynamic equilibrium, where transitions between clonogenic states are modulated by EV-mediated Wnt signaling. DNA methylation modulated SP–non-SP transitions and was correlated with the reciprocal expressions of Wnt signaling pathway agonist Wnt3a in SP cells and the antagonist secreted frizzled-related protein 4 in non-SP cells. Lymphoma SP cells exhibited autonomous clonogenicity and exported Wnt3a via small EVs to neighboring cells, thus modulating population equilibrium in the tumor. Of note, macrophages contribute to the proliferation of DLBCL cells. Macrophages do not maintain a fixed functional state; instead, they polarize into two discrete subtypes (M1 and M2), and this functional shift is directly dictated by the specific stimuli they encounter in their microenvironment. MiR-7e-5p carried by EVs can be transferred from DLBCL cells to M1 macrophages. A study by Lou X et al. [11] demonstrated that miR-7e-5p expression is significantly downregulated in highly aggressive high-grade follicular lymphoma (FL) and DLBCL. This downregulation reduces the transfer of miR-7e-5p via EVs, which in turn upregulates FasL expression and activates the apoptosis-related caspase signaling pathway in activated M1 macrophages. Mechanistically, DLBCL cell-derived EVs exert dual regulatory effects on macrophages: first, they enhance the expression of PGC-1β [6]; second, EVs containing neuron-specific enolase (NSE) disrupt the NF-κB pathway to induce macrophage polarization toward the M2 phenotype, while also promoting macrophage migration [7]. These combined effects drive DLBCL progression. Clinically, elevated NSE expression correlates with poor prognosis in DLBCL patients, particularly those with the non-germinal center B-cell (non-GCB) subtype [12,13]. However, Chen et al. demonstrated that EVs derived from DLBCL also exert anti-tumor effects, which are mediated by dendritic cells activated by these EVs [14].

### 2.2. Treatment

To date, the classical R-CHOP regimen remains the first-line standard of care for DLBCL, comprising rituximab, cyclophosphamide, doxorubicin, oncovin, and prednisone. In murine models, studies have demonstrated that EVs downregulate TNFAIP3 through miR-125b-5p, leading to decreased rituximab sensitivity in DLBCL cells and subsequent alterations in CD20 expression [3]. Interestingly, preclinical studies have implied a potential association between the therapeutic effects of this regimen and EVs. Specifically, in vitro and murine model studies have shown that DLBCL cells secrete small EVs loaded with doxorubicin and pixantrone, both of which are topoisomerase II inhibitors. Specifically, studies have demonstrated that DLBCL cells secrete small EVs loaded with doxorubicin and pixantrone, which are both classified as topoisomerase II inhibitors [8].

### 2.3. Drug Resistance

The production of small EVs relies on the ATP-binding cassette transporter A3 (ABCA3); when the expression of ABCA3 is suppressed, it leads to a higher accumulation of drugs within tumor cells. In DLBCL, tumor cells develop drug resistance via small EVs that encapsulate doxorubicin and pixantrone. Interestingly, indomethacin is able to boost the anticancer performance of these two drugs against DLBCL, and this beneficial effect is brought about by its ability to inhibit ABCA3 [15]. Anti-CD20 monoclonal antibodies are the cornerstone of DLBCL treatment, and suboptimal therapeutic efficacy is closely linked to CD20-mediated resistance. DLBCL cells evade rituximab activity via EVs, which drive the downregulation of CD20 expression in these cells. This process occurs as EV-transported microRNA-125b-5p modulates gene expression to achieve such a reduction [3]. Carbonic anhydrase 1 (CA1) exhibits elevated expression in patients with DLBCL, and it drives chemoresistance by activating the NF-κB and STAT3 signaling pathways [5].

### 2.4. EV-Derived Biomarkers for Monitoring

Due to their advantages of convenience, non-invasiveness, and lack of radiation exposure, EVs have been widely utilized in the diagnosis and therapeutic effect monitoring of some tumor diseases in recent years, representing the most prevalent clinical application of EVs at present. Rune Matthiesen et al. [16] conducted a comparative study involving 32 patients with newly diagnosed DLBCL and 15 age-matched healthy individuals. In this research, EVs were isolated from plasma samples via ultracentrifugation, followed by physical characterization using nanoparticle tracking analysis to assess features like particle size and concentration. For proteomic profiling, label-free quantitative mass spectrometry was employed to analyze the protein composition of these EVs. The results revealed that multiple EV-derived proteins—including immunoglobulin lambda constant 1 (IGLC1), immunoglobulin lambda-like polypeptide 5 (IGLL5), and proteasome subunit beta type-2 (PSMB2)—exhibited a significant correlation with patient survival outcomes. These proteins thus hold potential as prognostic biomarkers for DLBCL. Currently, the evaluation system for therapeutic efficacy monitoring predominantly relies on imaging modalities, including positron emission tomography–computed tomography (PET-CT), magnetic resonance imaging (MRI), computed tomography (CT), and ultrasound. With the in-depth advancement of research on EVs, these vesicles have been identified as viable indicators for efficacy assessment. Specifically, Feng Yuhua et al. demonstrated that the expression levels of miR-99a-5p and miR-125b-5p are significantly higher in chemotherapy-resistant patients than in chemotherapy-sensitive counterparts, with their respective area under the curve (AUC) values reaching 0.744 and 0.780 [17]. Nasrin Zare et al. have also reported consistent findings: compared to patients who responded effectively to chemotherapy or are currently undergoing chemotherapy, miR-155 expression is significantly elevated, while Let-7g expression is notably decreased in those with recurrent and refractory disease [18].

### 2.5. Future Perspectives

In vitro investigations have demonstrated that diminished miR-107 expression is linked to ECOG performance score, pathological staging, and elevated levels of IPI, LDH, and beta-2 microglobulin—all of which indicate an unfavorable clinical outcome [4]. A separate investigation documented that elevated IPI scores correspond with increased extracellular CA1 expression, hinting at a potential association between CA1 and the invasive properties of the disease [5]. These prognosis-associated EV-derived molecules could serve as promising targets for future therapeutic intervention and disease monitoring, offering new hope to patients with relapsed or refractory DLBCL and even paving the way for the development of novel breakthrough therapeutics similar to rituximab.

## 3. Burkitt’s Lymphoma (BL)

Burkitt’s lymphoma is a highly aggressive B-NHL and one of the most rapidly proliferating malignant tumors in humans. The overall incidence of this disease is extremely low, accounting for only 1–2% of all NHL cases. Children and adolescents are high-risk groups, with two additional incidence peaks in adults aged 40 and 75 years. From a typological perspective, endemic cases are concentrated in malaria-endemic regions and represent the most common pediatric malignancy in these areas. Sporadic cases show no obvious racial or ethnic predilection, while immunodeficiency-associated cases are more prevalent in HIV-infected individuals aged 40–45 years, accounting for nearly 40% of HIV-related lymphomas. Etiological factors vary by subtype: endemic cases are almost universally associated with Epstein–Barr virus (EBV) infection, and malaria epidemics exacerbate chromosomal breakage, further increasing disease risk. Twenty to thirty percent of sporadic cases are EBV-related, with a higher incidence in patients over 50 years old. The EBV detection rate in immunodeficiency-associated cases ranges from 25% to 40%, and these cases typically occur in HIV-infected individuals with relatively normal CD4 counts. Chemotherapy regimens modified from R-CHOP serve as the cornerstone of treatment for this disease. Children and young adults have a favorable prognosis, with a 5-year survival rate of 80–90%. In contrast, elderly patients may experience poorer treatment outcomes due to impaired immune function and potential comorbidities, and the treatment failure rate in adults can be as high as 35%.

### 3.1. EV-Mediated Pathogenesis

Small EVs serve as key mediators of intercellular communication in BL, driving oncogenesis through diverse cargo-dependent mechanisms. Epstein–Barr virus (EBV)-encoded latent membrane protein 1 (LMP1) is a critical oncogenic cargo: it is packaged into small EVs via a CD63-dependent pathway, with CD63 essential for LMP1’s perinuclear localization and exosomal secretion [19]. These LMP1-containing small EVs enhance EV production and promote malignant cell growth, migration, and invasion by activating NF-κB, PI3K/Akt, and MAPK/ERK signaling pathways [19,20]. Notably, CD63 also limits LMP1-induced noncanonical NF-κB activation, balancing oncogenic signaling [19]. Exosomal microRNAs (miRNAs) further contribute to lymphoma pathogenesis. EBV-positive Burkitt lymphoma (BL) Raji cells secrete small EVs carrying miR-155, which targets von Hippel–Lindau (VHL) to upregulate HIF-1α and VEGF-A, inducing angiogenesis in recipient cells [21]. Raji-derived small EVs also transfer miR-106a, which inhibits Beclin1 to suppress autophagy and apoptosis, promoting lymphoma cell proliferation [22]. Additionally, during EBV activation, the BMRF1-encoded EA-D protein accumulates in polymorphic (58, 50, 48, 44 kDa) phosphorylated/dephosphorylated forms, localizing to intracellular vesicles and extracellular particles in BL cell lines [23]. miR-26a-5p, identified as a stable reference miRNA for exosomal miRNA quantification, highlights the potential of exosomal miRNAs as consistent pathogenic mediators in pediatric hematological malignancies [24].

### 3.2. Treatment

Emerging therapies focus on disrupting EV-mediated oncogenic pathways. CD63, as a critical regulator of LMP1 exosomal trafficking, represents a promising target to block LMP1’s pro-tumorigenic signaling [19]. Inhibiting miR-155 in EBV-positive BL small EVs reduces VEGF-A expression and angiogenesis, supporting miR-155 inhibitors as potential therapeutics [21]. Similarly, silencing miR-106a or overexpressing Beclin1 reverses EV-induced suppression of autophagy and apoptosis, restoring anti-tumor responses [22]. Disrupting exosomal biogenesis with GW4869 also inhibits small EV secretion, attenuating lymphoma cell growth [22]. These strategies leverage small EV biology to target key oncogenic cargo and signaling cascades.

### 3.3. Drug Resistance

While direct evidence of EV-mediated drug resistance is limited, related pathways suggest critical links. LMP1-induced activation of NF-κB and PI3K/Akt, modulated by CD63-dependent exosomal trafficking, is associated with treatment resistance in EBV-associated malignancies [19]. MiR-155-mediated VHL/HIF-1α pathway activation may enhance tumor microenvironment adaptation, contributing to resistance [21]. Furthermore, exosomal miR-106a’s suppression of autophagy could reduce sensitivity to therapies targeting autophagic cell death [22]. The stability of exosomal miRNAs like miR-26a-5p across isolation methods suggests they may serve as resistance-related biomarkers, though further studies are needed to confirm direct resistance mechanisms [24].

### 3.4. EV-Derived Biomarkers for Monitoring

Small EVs offer noninvasive biomarkers for lymphoma diagnosis and progression monitoring. miR-26a-5p exhibits stable expression in plasma small EVs of healthy donors and pediatric lymphoma patients, making it a reliable reference for qRT-PCR normalization in exosomal miRNA quantification [24]. The polymorphic EA-D protein, with its phosphorylation dynamics, serves as a marker for EBV activation in BL cell lines [23]. For EBV-positive BL, exosomal miR-155 and its downstream target VEGF-A can monitor angiogenic activity [21]. Additionally, miR-106a and Beclin1 expression levels in small EVs or tumor tissues reflect lymphoma progression, as their dysregulation correlates with impaired autophagy and apoptosis [22]. These biomarkers enable liquid biopsy-based non-invasive disease monitoring in clinical settings.

### 3.5. Future Perspectives

Future research may focus on translating EV-related mechanistic insights from preclinical models into early clinical exploration and validation, as targeting CD63-mediated LMP1 trafficking or the miR-155/miR-106a pathways has the potential to disrupt oncogenic signaling [19,21,22]. Developing EV-based delivery systems for miRNA inhibitors or Beclin1 overexpression constructs may restore anti-tumor autophagy and apoptosis [22]. Optimizing exosomal biomarker panels—combining miR-26a-5p, EA-D, and miR-155—could improve diagnostic accuracy and treatment response monitoring [21,23,24]. Further studies are needed to validate CD63 and LMP1 as therapeutic targets in EBV-associated lymphomas and explore EV-mediated crosstalk between lymphoma cells and the tumor microenvironment [19,20]. Standardizing small EV isolation methods will also enhance the reliability of exosomal biomarkers for clinical use [19,24].

In summary, small EVs drive lymphoma pathogenesis through viral proteins, miRNAs, and other cargo, while offering novel therapeutic targets and biomarkers. Harnessing small EV biology holds great potential for improving lymphoma treatment, monitoring, and patient outcomes.

## 4. Follicular Lymphoma (FL)

Follicular lymphoma ranks as the second most prevalent subtype of lymphoma in Western Europe, boasting an annual incidence of 5 cases per 100,000 individuals. In contrast, the incidence is notably lower in Asia and Africa, ranging from approximately 0.5 to 1 case per 100,000 people. Within China, FL accounts for roughly 8% to 23% of all NHL cases, with an incidence rate mirroring that of Asia and Africa (0.5–1 per 100,000). As the most common indolent NHL, FL predominantly affects elderly populations, with around 70% of patients diagnosed at an advanced stage (III or IV). Despite the late-stage presentation for many, the overall prognosis remains favorable: the 5-year survival rate exceeds 80%, and numerous patients achieve long-term remission following standardized treatment—some even maintaining disease-free survival for over a decade. However, recurrence remains a persistent and challenging issue: a subset of patients experiences multiple relapses, with treatment efficacy gradually declining with each recurrence episode.

### 4.1. EV-Mediated Pathogenesis

Small EVs play pivotal roles in the pathogenesis of hematological malignancies by mediating intercellular communication and reshaping the tumor microenvironment. In EBV-positive classic Hodgkin lymphoma (cHL), follicular dendritic cells (FDCs) in reactive germinal centers (GCs) capture latent membrane protein-1 (LMP1) from peri-follicular EBV-infected cells via small EVs, as evidenced by the co-localization of LMP1 with exosomal marker CD63 and FDC marker CD21 [25]. This LMP1 accumulation in FDCs contributes to an immunosuppressive microenvironment by inhibiting immune cell proliferation [25]. In FL, B-cell receptor (BCR) stimulation of the FL DOHH2 cell line induces polarization of CD63+ MHC class II compartments and enhances small EV secretion; the BCR also targets its bound antigen to these vesicles to facilitate antigen transfer [26]. FL-derived small EVs are internalized by bone marrow mesenchymal stromal cells (BM-MSCs) through clathrin- and caveolin-dependent endocytosis, activating TGF-β-dependent and independent pathways (e.g., SMAD2/3, p38, STAT6). This activation upregulates hematopoietic stem cell niche factors such as CXCL12 and angiopoietin-1, promoting FL cell survival and quiescence [27]. Additionally, FL cells secrete microvesicles (MVs) containing IL-31, which activates STAT1/3, ERK1/2, and Akt pathways to enhance tumor cell proliferation, with higher IL-31/IL-31RA expression in high-grade FL indicating a role in disease progression [6].

### 4.2. Therapy

Small EVs hold potential as therapeutic tools and targets in hematological malignancies. Tumor-derived small EVs can transfer tumor-associated antigens to antigen-presenting cells, inducing antigen-specific anti-tumor immune responses, which is promising for immunotherapy in FL where shared tumor rejection antigens are lacking [26]. Targeting the crosstalk between FL-derived small EVs and BM-MSCs is a viable strategy, as inhibiting EV-mediated stromal polarization could disrupt the supportive niche for malignant B cells [27]. The TGF-β signaling pathway activated by FL small EVs in BM-MSCs also represents a therapeutic target, as TGF-β inhibitors may reverse stromal cell reprogramming [27]. Furthermore, blocking IL-31-containing MVs in FL could abrogate autocrine/paracrine proliferation signals, providing a novel treatment avenue [28].

### 4.3. Drug Resistance

Small EVs contribute to drug resistance by inducing a quiescent phenotype and modifying the tumor niche. FL-derived small EVs prime BM-MSCs to support FL cells with reduced proliferation (downregulated MKI67 and CDK1) and enhanced quiescence, a state associated with decreased sensitivity to chemotherapeutic agents targeting dividing cells. This quiescent phenotype mimics BM-infiltrating FL cells, which exhibit lower cytological grade and proliferation. Additionally, small EVs can protect malignant B cells from immunotherapy by binding therapeutic anti-CD20 antibodies, as reported in aggressive B-cell lymphoma, suggesting a similar mechanism may operate in FL. The upregulation of CXCL12 by EV-primed BM-MSCs also promotes FL cell adhesion and survival, further contributing to treatment resistance [27].

### 4.4. EV-Derived Biomarkers for Monitoring

Exosomal microRNAs (miRNAs) serve as non-invasive biomarkers for diagnosing and monitoring hematological malignancies. Serum exosomal miR-155 is significantly elevated in chronic lymphocytic leukemia (CLL), acute myeloid leukemia (AML), and Waldenström’s macroglobulinemia (WM), while reduced in myelodysplastic syndrome (MDS) and multiple myeloma (MM). Notably, exosomal miR-155 levels do not differ significantly in FL, DLBCL, or Hodgkin lymphoma (HL) compared to controls, highlighting its disease-specific utility [29].

### 4.5. Future Perspectives

Future research should focus on unraveling the precise cargo of small EVs in different hematological malignancies to identify key mediators of tumor progression. For example, characterizing the miRNA and protein profiles of FL small EVs could reveal additional therapeutic targets beyond TGF-β and CXCL12 [27]. Optimizing small EV isolation and detection methods will enhance the clinical applicability of exosomal biomarkers like miR-155, enabling early diagnosis and real-time monitoring of treatment response [29]. Developing strategies to block small EV release or uptake (e.g., targeting endocytosis pathways) may disrupt the tumor-stroma crosstalk in FL and cHL [25,27]. Additionally, engineering small EVs to deliver therapeutic agents (e.g., siRNA, chemotherapeutic drugs) directly to malignant B cells or stromal cells could improve treatment efficacy while minimizing off-target effects. Furthermore, well-designed clinical studies are needed to validate these exosomal biomarkers and therapeutic strategies, thereby translating preclinical findings into personalized medicine for patients with B-cell hematological malignancies.

## 5. Mantle Cell Lymphoma (MCL)

The global annual incidence of mantle cell lymphoma is approximately 0.5–0.8 per 100,000 individuals, representing 3–10% of all NHL cases. The median age at onset ranges from 60 to 70 years, with patients aged ≥65 years accounting for over 60% of cases. MCL shows a significant male predominance, with a male-to-female ratio of 2–4:1. Around 80% of patients are diagnosed at an advanced stage (Stage III/IV) upon initial presentation. Nearly 95% of MCL patients harbor the t(11; 14) (q13; q32) chromosomal translocation, which drives the overexpression of Cyclin D1—a key pathogenic feature of the disease. Treatment strategies are tailored to the patient’s physical status, with chemotherapy or oral targeted agents serving as the mainstay of therapy. However, 10–15% of patients exhibit primary drug resistance (e.g., due to TP53 mutations or blastoid variants), failing to respond to first-line immunochemotherapy or initial Bruton’s tyrosine kinase (BTK) inhibitor treatment. These patients have a median overall survival of only 1–2 years, with virtually no curative options available. Even among patients who achieve complete remission (CR) with initial treatment, most experience disease recurrence within 2–5 years. Notably, the treatment response rate tends to decline progressively with each subsequent recurrence, posing a major clinical challenge.

### 5.1. EV-Mediated Pathogenesis

Small EVs play a critical role in the progression of MCL and multiple myeloma (MM) by mediating intercellular communication. MCL-derived small EVs are preferentially internalized by B-lymphocytes (both malignant and healthy) rather than T-lymphocytes, NK cells, or stromal cells, via a cholesterol/lipid raft-dependent pathway that is independent of clathrin and caveolin-1 [30]. In MM and MCL, small EVs carry HSP70-1, which forms an autocatalytic loop: extracellular HSP70-1 is taken up by tumor cells, stimulating endogenous HSP70-1 promoter activity to drive proliferation [31]. MCL small EVs are detectable in patient serum, with higher levels correlating with increased white blood cell counts, confirming their systemic role [30].

### 5.2. Therapy

Small EVs serve as both therapeutic targets and delivery vehicles. IVIgG contains anti-HSP70-1 IgG that binds extracellular HSP70-1, blocking its EV-mediated uptake and enhancing the efficacy of HSP90 inhibitors (e.g., AUY922) and proteasome inhibitors (e.g., bortezomib) [31]. In vivo, the IVIgG-bortezomib combination outperforms single agents in suppressing MM tumor growth. MCL small EVs’ natural tropism for B-lymphocytes enables targeted delivery of therapeutics like cyclin D1 siRNA, minimizing off-target effects [30].

### 5.3. Drug Resistance

Small EVs mediate acquired resistance: bortezomib-resistant MM cell-derived small EVs transfer HSP70-1 to sensitive cells, increasing HSP70-1 and p53 levels to counteract drug effects [31]. IVIgG blocks small EV uptake by ~50%, preventing resistance transmission and restoring drug sensitivity [31]. MCL small EVs’ high affinity for malignant B-cells also facilitates intercellular transfer of survival signals, contributing to resistance [30].

### 5.4. EV-Derived Biomarkers for Monitoring

Small EVs are valuable biomarkers. Proteomic analysis of malignant B-cell microparticles identified CD148 as an MCL-specific biomarker [32]. Flow cytometry validation in 158 patients showed CD148 MFI was significantly higher in MCL (mean = 613) than CLL (189), SLL (209), or controls (168) (*p* < 0.0001). A CD148 MFI ≥ 2-fold that of CLL/SLL enables MCL diagnosis with 91% specificity and 78% sensitivity [32]. Circulating MCL-derived small EVs expressing CD81, CD63, and CD20 in patient serum also serve as valuable non-invasive clinical monitoring tools [30].

### 5.5. Future Perspectives

Key research directions include developing HSP70-1 monoclonal antibodies as cost-effective alternatives to IVIgG, optimizing EV-based targeted drug delivery systems, and expanding exosomal biomarker panels [31]. In addition, further in-depth research into the cargo of small EVs and rigorous clinical validation of these targeting strategies are urgently needed. CD148’s combination with cyclin D1 could improve MCL diagnostic accuracy, guiding aggressive therapies [32]. Small EV research holds great promise for advancing the preclinical development of precision medicine in B-cell malignancies, with potential clinical translation pending further large-scale studies.

## 6. Common Mechanisms Across B-NHL Subtypes

While each lymphoma subtype has unique characteristics, several EV-mediated mechanisms are shared across B-NHLs, warranting a comparative synthesis:

NF-κB Activation: A recurring theme in DLBCL, BL, and MCL is the activation of the NF-κB pathway by EV cargo (e.g., LMP1 in BL, NSE in DLBCL), driving proliferation and survival.
MiRNA-Mediated Regulation: MiRNAs such as miR-155 and miR-125b-5p are frequently dysregulated in EVs across subtypes, contributing to drug resistance and immune evasion.
Microenvironment Remodeling: EVs consistently function to reshape the tumor microenvironment, whether by polarizing macrophages in DLBCL, activating stromal cells in FL, or transferring heat shock proteins in MCL.
Recognizing these commonalities suggests that therapeutic strategies targeting universal EV biogenesis or uptake pathways (e.g., CD63 inhibition, heparin blockade) could have broad applicability across multiple B-NHL subtypes.

## 7. Critical Challenges and Future Perspectives

Despite the promising potential of EVs in B-NHL, several critical barriers hinder their immediate clinical translation. It is imperative to address these limitations to advance the field.

### 7.1. Technical Limitations: Isolation, Standardization and Heterogeneity of EVs

The integrated evidence from recent studies and guidelines reveals that extracellular vesicle (EV) research continues to be constrained by three interrelated technical challenges: (1) limitations of current isolation methods, (2) lack of harmonized standardization, and (3) inherent heterogeneity of EV populations. These factors collectively impair reproducibility, comparability, and clinical translatability.

#### 7.1.1. Isolation Methods: Compromises Among Yield, Purity, and Structural Integrity

No single isolation method currently satisfies the simultaneous demands of high yield, high purity, and preserved biological activity. Comparative analyses show clear trade-offs: Precipitation methods (e.g., PEG, commercial polymer kits) deliver high particle yield, making them suitable for limited sample volumes, but they co-isolate abundant non-vesicular components such as lipoproteins, protein aggregates, and nucleic acid complexes, reducing specificity [33,34,35]. Size exclusion chromatography (SEC) provides superior specificity and efficient removal of soluble plasma proteins, but its yield is relatively low, and it cannot fully separate EVs from similarly sized lipoproteins [33,34]. Ultracentrifugation is widely accessible and commonly used, yet it is time-consuming, operator-dependent, and risks mechanical damage to vesicles. Density gradient ultracentrifugation improves purity but adds complexity and time [33,34]. Immunoaffinity capture enables isolation of specific subpopulations but is limited by low yield, high cost, and potential loss of function during elution [34,35]. Microfluidic, asymmetric flow field-flow fractionation (AF4), and related advanced techniques offer high resolution and the possibility of integrated processing, but their complexity, need for specialized instrumentation, and limited validation across diverse sample types currently restrict broad adoption [34,36]. For example, in plasma EV studies, precipitation achieved up to 50 × 10^9^ particles/mL but detected only 15 of the top 100 EV markers, whereas SEC recovered 62 markers with >90% depletion of albumin and IgG, highlighting the yield–purity trade-off [33]. In urinary EV isolation, differential velocity centrifugation (DC) gave the most repeatable concentration and size measurements, but required multiple steps and ultracentrifugation, while SiC and PEG methods were faster but less consistent [36]. MISEV2023 explicitly discourages opaque commercial kits due to their hidden separation principles, which hinder reproducibility and cross-study comparison [35].

#### 7.1.2. Standardization: From Sample Handling to Reporting

Lack of standardization remains a principal obstacle to reproducibility in EV research: Pre-analytical variability—including differences in sample collection timing, storage temperature, freeze–thaw cycles, and preprocessing centrifugation—significantly alters EV yield, size distribution, and molecular profiles [33]. For instance, first morning urine samples consistently contained higher EV counts than afternoon or evening collections [36]. Analytical variability stems from instrument-specific settings: NTA, DLS, and flow cytometry can yield divergent results depending on camera sensitivity, detection thresholds, syringe pump speeds, and focus positions. Fixed protocols and pooled technical replicates are recommended to mitigate this [36]. Reporting inconsistencies persist across the literature. Essential methodological details—exact centrifugal forces, rotor types, column dimensions, incubation times, and buffer compositions—are frequently omitted, hampering replication [34,35]. Quality assessment remains rudimentary in many studies, relying on total protein/particle ratios or a handful of generic markers. MISEV2023 proposes a five-component framework for evaluating EV protein content (membrane-associated proteins, cytosolic proteins, common contaminants, intracellular organelle components, and co-isolated secreted proteins), yet uptake is incomplete [35]. These standardization gaps produce a fragmented data landscape where comparability across laboratories and multicenter studies is compromised [33,34,35,36].

#### 7.1.3. Heterogeneity: Physical, Molecular, and Functional

Complexity EV heterogeneity fundamentally limits precise isolation and functional attribution: Physical overlap with non-vesicular particles such as lipoproteins and protein aggregates complicates purification. Marker proteins like CD9, CD63, and CD81 are detectable in both EV-rich and EV-depleted fractions, implying that their mere presence does not confirm vesicle encapsulation [33]. Biogenetic and size heterogeneity blur classical boundaries between “exosomes” and “microvesicles.” MISEV2023 advises caution with these terms unless endosomal origin is confirmed, yet they remain widely and interchangeably used [34,35]. Molecular diversity in protein, lipid, and nucleic acid composition varies with parental cell type, physiological state, and microenvironment, meaning that even within the same subclass, different subpopulations can carry distinct cargos and exert different effects [34,36]. Functional pleiotropy is evident in both in vitro and in vivo settings, where identical EV preparations can trigger divergent responses in different recipient cells, depending on local context [35]. In urinary EV studies, for example, microvesicles were more polydisperse than exosomes, and both fractions overlapped in size. Single-vesicle optical redox ratio measurements revealed inter- and intra-individual metabolic variability, supporting the use of personalized reference intervals for physical parameters but population-based intervals for functional readouts [36].

#### 7.1.4. Implications for Clinical Translation

The combined technical limitations have direct consequences for the development of EV-based diagnostics and therapeutics: Product variability in clinical trials, such as those involving MSC-derived EVs, undermines the reliability of safety and efficacy assessments [35]. Ambiguous functional assignment caused by co-isolated contaminants and subtype overlap makes it difficult to link specific biological activities to well-defined EV populations [33,34]. Regulatory uncertainty persists in the absence of a widely accepted gold-standard method for producing clinical-grade EVs, although initial steps toward harmonization of criteria for MSC- and iPSC-derived small EVs have been proposed [35].

### 7.2. Limitations in Study Design: Small Sample Sizes and Lack of Stratification

A prominent limitation across the majority of current EV-related B-NHL research lies in the small sample sizes of patient cohorts employed in biomarker discovery, mechanistic validation, and therapeutic efficacy exploration. Most studies cited in this review, including those identifying EV-derived protein biomarkers (e.g., IGLC1, CD148) and miRNA biomarkers (e.g., miR-125b-5p, miR-155), rely on relatively small patient groups (typically *n* < 50, and in some cases fewer than 30 participants). This small-scale sampling severely restricts the statistical power of the research, leading to an increased risk of type I and type II errors, and making it difficult to detect subtle but biologically meaningful correlations between EV cargo profiles and clinical outcomes (e.g., prognosis, treatment response, drug resistance). Moreover, the limited sample size undermines the generalizability of study findings, as the results may be skewed by individual patient heterogeneity and cannot be reliably extrapolated to the broader B-NHL patient population, including diverse ethnic, age, and clinical subpopulations.

A second critical design flaw is the lack of rigorous clinical stratification in patient enrollment and data analysis. B-NHL is a highly heterogeneous disease, with distinct subtypes (DLBCL, BL, FL, MCL) exhibiting unique clinical characteristics, genetic backgrounds, and treatment responses; even within a single subtype (e.g., DLBCL’s GCB and non-GCB subtypes), there is significant molecular and clinical variability. Additionally, key clinical variables—such as disease stage, prior treatment history, presence of high-risk genetic mutations (e.g., TP53 in MCL), EBV status (e.g., in BL and FL), and the International Prognostic Index (IPI) score—exert a profound impact on EV secretion, cargo composition, and the biological function of EVs in tumor progression. However, many current studies fail to stratify patient cohorts by these critical factors, or conduct subgroup analyses with sufficient statistical power. This lack of stratification leads to the confounding of study results: the observed associations between specific EV markers and clinical endpoints may be masked or misattributed, rather than reflecting the true biological role of EVs in distinct B-NHL subpopulations. For example, studies investigating EV-derived miR-155 as a biomarker may fail to account for EBV status in BL patients, where EBV-driven miR-155 expression is a key pathogenic factor, leading to inconsistent findings across mixed EBV-positive and EBV-negative cohorts.

Further, the lack of stratification extends to control group design in many studies. Control groups are often limited to healthy age-matched individuals, with few studies including disease controls (e.g., patients with other B-cell hematological malignancies, non-malignant lymphoproliferative disorders) or matched B-NHL patients with different treatment responses (e.g., complete remission vs. refractory/relapsed). This gap prevents researchers from distinguishing EV biomarkers or functional effects that are specific to B-NHL (or a particular subtype) from those that are non-specific to malignant B-cell disorders or general inflammatory states, reducing the clinical utility of identified EV targets and biomarkers.

Collectively, the small sample sizes and lack of systematic stratification in current study designs hinder the translation of preclinical and early clinical EV research into clinically actionable tools for B-NHL. These design limitations result in inconsistent findings across the literature, make it difficult to validate potential EV-based biomarkers and therapeutic targets, and delay the development of personalized EV-related diagnostic and treatment strategies for B-NHL patients. Addressing these flaws will require large-scale, multicenter studies with standardized patient stratification by disease subtype, molecular characteristics, clinical stage, and treatment history, as well as well-designed control groups and sufficient statistical power to detect subtype- and context-specific EV-mediated effects.

### 7.3. Translational Limitations: From Preclinical Models to Clinical Application

The translation of EV-related research in B-NHL from preclinical models to clinical practice is hampered by discrepancies between experimental systems and human disease, insufficient clinical validation, technical barriers to therapeutic development, and regulatory uncertainties, which collectively slow the conversion of promising preclinical findings into clinical tools [1,35].

#### 7.3.1. Preclinical Model Limitations

Preclinical EV studies rely primarily on cell line cultures and immunodeficient murine models, which fail to recapitulate the human B-NHL tumor microenvironment (TME) and systemic disease complexity [6,7]. Immunodeficient mice lack functional immune systems, precluding evaluation of critical EV-mediated immunomodulatory effects (e.g., DLBCL EV-induced M2 macrophage polarization [7]). Cell line-derived EVs have homogeneous cargo profiles, unlike the highly heterogeneous EVs from B-NHL patients, which are shaped by genetic background, disease stage, and treatment history [33]. Additionally, preclinical models do not account for in vivo EV biodistribution, clearance, or off-target accumulation—factors that reduce the efficacy of EV-based drug delivery systems in humans [30,34].

#### 7.3.2. Inadequate Clinical Validation of EV Biomarkers

EV-derived biomarkers (e.g., CD148 for MCL [32], miR-125b-5p for DLBCL [17]) lack large-scale, multicenter clinical validation. Most studies use small cohorts (*n* < 50) with insufficient statistical power, limiting the generalizability of findings [16,33]. Confounding clinical factors (e.g., comorbidities, infections) and non-standardized EV isolation/quantification methods (e.g., ultracentrifugation vs. precipitation [33,36] further cause inconsistent biomarker detection, even for well-characterized markers like miR-155 [29]. Poor adherence to MISEV2023 guidelines exacerbates these issues, hindering the integration of EV biomarkers into routine clinical practice [35].

#### 7.3.3. Barriers to EV-Targeted Therapeutic Development

Developing EV-targeted therapeutics faces critical specificity and scalability challenges. Inhibiting universal EV biogenesis pathways (e.g., CD63 [19], HSP70-1 [31]) disrupts physiological EV functions (e.g., dendritic cell-mediated antigen presentation [14]), leading to off-target immune impairment. Clinical-grade EV manufacturing is also costly and time-consuming, with EV preparations sensitive to temperature, pH, and freeze–thaw cycles that compromise structural and cargo integrity [33,34]. Engineered EV drug delivery systems, though effective in vitro [30], lack optimized storage and administration protocols for clinical use [34].

#### 7.3.4. Regulatory and Clinical Trial Hurdles

The evolving regulatory landscape for EV-based products creates uncertainty, as the FDA/EMA lack specific guidelines for evaluating the safety, efficacy, and quality of EV diagnostics/therapeutics [35]. EV biomarker validation is further hindered by the absence of standardized reference materials [33]. Clinical trials for EV-targeted therapies suffer from poor patient stratification—no established biomarkers predict treatment response in heterogeneous B-NHL subtypes—and unclear optimal dosages, administration routes, and combinations with standard regimens (e.g., R-CHOP). Only a small fraction of ongoing B-NHL clinical trials focus on EV-targeted strategies, reflecting slow translational progress [1].

### 7.4. Underexplored Research Avenues and Reporting Norms

Beyond the aforementioned translational barriers, two additional aspects warrant attention for the rigorous development of EV research in B-NHL. First, the link between EVs and systemic metabolic reprogramming in B-NHL remains underexplored. While metabolic dysregulation is a known feature of cancer and a risk factor for lymphoma, current evidence for EV-mediated metabolic crosstalk (e.g., lipid transfer, glycolysis modulation) between lymphoma cells and the tumor microenvironment is scarce, with most reported EV effects being immunomodulatory or signal-transductive. Future research should prioritize investigating this unaddressed mechanistic link to expand the understanding of EV function in B-NHL pathogenesis. Second, balanced and cautious language is essential when interpreting the clinical potential of EV-based strategies. Although preclinical mechanistic data support associations between EVs and the efficacy of standard regimens such as R-CHOP, these claims require further clinical validation. The field must move beyond descriptive characterizations of EV cargo to focus on functional interventions that demonstrate clear, clinically meaningful benefits for B-NHL patients.

In conclusion, EVs are inextricably tied to lymphoma biology, influencing pathogenesis, treatment efficacy, and resistance. Looking ahead, addressing methodological inconsistencies and conducting robust clinical trials will be essential to harness the full potential of EVs in the management of B-NHL.

## 8. Summary

**Figure 2:**
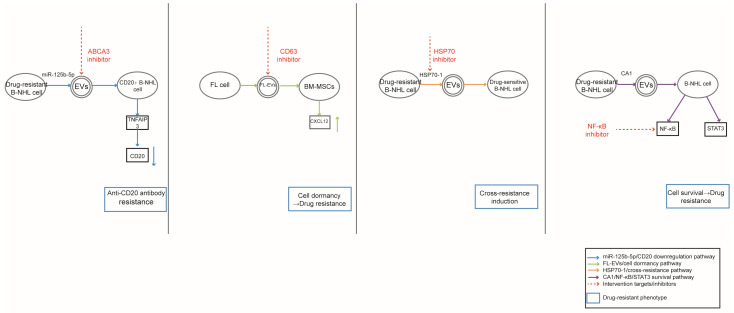
EV-mediated drug resistance mechanisms in B-NHL.

This review systematically summarizes the roles of EVs in four major B-NHL subtypes: diffuse large B-cell lymphoma (DLBCL), Burkitt lymphoma (BL), follicular lymphoma (FL), and mantle cell lymphoma (MCL). In terms of pathogenesis, EVs mediate intercellular crosstalk by shuttling diverse cargoes, including Wnt3a, miR-155, Epstein–Barr virus (EBV)-encoded LMP1, and HSP70-1. These cargoes activate downstream signaling pathways (e.g., NF-κB, PI3K/Akt) to drive tumor cell proliferation, invasion, immune suppression, and angiogenesis, while also regulating the dynamic equilibrium of tumor cell subpopulations and macrophage polarization. These cargoes activate downstream signaling pathways (e.g., NF-κB, PI3K/Akt) to drive tumor cell proliferation, invasion, immune suppression, and angiogenesis, while also regulating the dynamic equilibrium of tumor cell subpopulations and macrophage polarization. In clinical practice, the classic R-CHOP regimen remains the first-line standard of treatment for most B-NHL subtypes, and preclinical and early clinical evidence points to a potential association between EVs and the therapeutic efficacy of this regimen in specific B-NHL subtypes. Emerging therapeutic strategies are being explored to target EV-mediated oncogenic pathways—for instance, inhibiting CD63 to block LMP1 secretion or engineering EVs as potential targeted drug delivery vehicles in preclinical models. In drug resistance, EVs have been shown to contribute to drug resistance in preclinical studies by reducing the expression of therapeutic targets (e.g., CD20), promoting tumor cell quiescence, transferring resistance-related molecules (e.g., HSP70-1), and activating pro-survival signaling pathways in specific B-NHL subtypes, which affects the efficacy of chemotherapy and targeted drugs (Figure 2). For clinical monitoring, EV-derived proteins (e.g., IGLC1, CD148) and miRNAs (e.g., miR-99a-5p, miR-125b-5p) have shown preliminary potential as non-invasive biomarkers for diagnosing specific B-NHL subtypes, evaluating prognosis, and monitoring treatment response and recurrence in small cohort studies. The table below summarizes this article (Table 1).

In conclusion, current preclinical and early clinical evidence indicates that EVs are closely implicated in B-NHL pathogenesis, as they modulate core disease processes including tumor progression, therapeutic response, and the development of drug resistance in specific B-NHL subtypes. Looking ahead, the clinical translation and application of EVs in B-NHL represent a promising research direction, and their potential clinical utility may be further expanded with additional large-scale clinical validation in the coming years. Future research on EVs in B-NHL must prioritize the standardization of EV isolation, characterization and biomarker detection protocols in accordance with MISEV2023 guidelines, and conduct large-scale, multicenter, stratified clinical studies to validate the reproducibility of EV-derived biomarkers. Only by addressing these critical limitations can the full clinical potential of EVs in the diagnosis, treatment and monitoring of B-NHL be realized.

## Figures and Tables

**Table 1:** Different EVs exert distinct effects on the occurrence and development of lymphoma.

Research Classification of EVs’ Effects on B-NHL	Specific Content
Overall Functional Positioning of EVs in B-NHL	As key mediators of intercellular communication, EVs are involved in the pathogenesis, treatment efficacy regulation, drug resistance development, and clinical monitoring of B-NHL (DLBCL, BL, FL, MCL).
Pathogenesis (EV-Mediated)	1. Carry cargoes (Wnt3a, miR-155, EBV-LMP1, HSP70-1, etc.); 2. Activate downstream signaling pathways (NF-κB, PI3K/Akt, etc.); 3. Promote tumor proliferation, invasion, immune suppression, angiogenesis, and regulate the dynamic balance of tumor cell subpopulations as well as macrophage polarization.
Treatment Relevance	1. The efficacy of the classic R-CHOP regimen is associated with EVs; 2. Emerging directions: Inhibit EV-mediated oncogenic pathways (e.g., inhibit CD63 to block LMP1 secretion) and use EVs as targeted drug delivery vehicles.
Drug Resistance Mechanisms	1. Reduce the expression of therapeutic targets (e.g., EV-mediated miR-125b-5p downregulates CD20); 2. Promote tumor cell quiescence; 3. Transfer drug resistance-related molecules (e.g., HSP70-1); 4. Activate pro-survival signaling pathways.
Clinical Monitoring (EV-Derived Biomarkers)	1. Protein markers: IGLC1, IGLL5, PSMB2 (DLBCL), CD148 (MCL), EA-D (BL); 2. miRNA markers: miR-99a-5p, miR-125b-5p (DLBCL), miR-26a-5p, miR-155 (BL); 3. Advantages: Non-invasive, radiation-free, applicable for diagnosis, prognosis evaluation, and monitoring of treatment response/recurrence.

## Data Availability

No new data were created or analyzed in this study.

## References

[1] Silkenstedt E., Salles G., Campo E., Dreyling M. (2024). B-cell non-Hodgkin lymphomas. Lancet.

[2] Théry C., Witwer K.W., Aikawa E., Alcaraz M.J., Anderson J.D., Andriantsitohaina R., Antoniou A., Arab T., Archer F., Atkin-Smith G.K. (2018). Minimal information for studies of extracellular vesicles 2018 (MISEV2018): A position statement of the International Society for Extracellular Vesicles and update of the MISEV2014 guidelines. J. Extracell. Vesicles.

[3] Zhang L., Zhou S., Zhou T., Li X., Tang J. (2021). Potential of the tumor-derived extracellular vesicles carrying the miR-125b-5p target TNFAIP3 in reducing the sensitivity of diffuse large B cell lymphoma to rituximab. Int. J. Oncol..

[4] Liu J., Han Y., Hu S., Cai Y., Yang J., Ren S., Zhao Y., Lu T., Zhou X., Wang X. (2021). Circulating Exosomal MiR-107 Restrains Tumorigenesis in Diffuse Large B-Cell Lymphoma by Targeting 14-3-3η. Front. Cell Dev. Biol..

[5] Feng Y., Zhong M., Tang Y., Liu X., Liu Y., Wang L., Zhou H. (2020). The Role and Underlying Mechanism of Exosomal CA1 in Chemotherapy Resistance in Diffuse Large B Cell Lymphoma. Mol. Ther. Nucleic Acids.

[6] Liu W., Zhu M., Wang H., Wang W., Lu Y. (2019). Diffuse large B cell lymphoma-derived extracellular vesicles educate macrophages to promote tumours progression by increasing PGC-1β. Scand. J. Immunol..

[7] Zhu M.-y., Liu W.-j., Wang H., Wang W.-d., Liu N.-w., Lu Y. (2019). NSE from diffuse large B-cell lymphoma cells regulates macrophage polarization. Cancer Manag. Res..

[8] Koch R., Aung T., Vogel D., Chapuy B., Wenzel D., Becker S., Sinzig U., Venkataramani V., von Mach T., Jacob R. (2016). Nuclear Trapping through Inhibition of Exosomal Export by Indomethacin Increases Cytostatic Efficacy of Doxorubicin and Pixantrone. Clin. Cancer Res..

[9] Aung T., Chapuy B., Vogel D., Wenzel D., Oppermann M., Lahmann M., Weinhage T., Menck K., Hupfeld T., Koch R. (2011). Exosomal evasion of humoral immunotherapy in aggressive B-cell lymphoma modulated by ATP-binding cassette transporter A3. Proc. Natl. Acad. Sci. USA.

[10] Koch R., Demant M., Aung T., Diering N., Cicholas A., Chapuy B., Wenzel D., Lahmann M., Güntsch A., Kiecke C. (2014). Populational equilibrium through exosome-mediated Wnt signaling in tumor progression of diffuse large B-cell lymphoma. Blood.

[11] Lou X., Fu J., Zhao X., Zhuansun X., Rong C., Sun M., Niu H., Wu L., Zhang Y., An L. (2020). MiR-7e-5p downregulation promotes transformation of low-grade follicular lymphoma to aggressive lymphoma by modulating an immunosuppressive stroma through the upregulation of FasL in M1 macrophages. J. Exp. Clin. Cancer Res..

[12] Wang L., Liu P., Geng Q., Chen X., Lv Y. (2011). Prognostic significance of neuron-specific enolase in patients with diffuse large B-cell lymphoma treated with rituximab-based immunochemotherapy. Leuk. Lymphoma.

[13] Wang L., Liu P., Chen X., Geng Q., Lu Y. (2011). Serum neuron-specific enolase is correlated with clinical outcome of patients with non-germinal center B cell-like subtype of diffuse large B-cell lymphoma treated with rituximab-based immunochemotherapy. Med. Oncol..

[14] Chen Z., You L., Wang L., Huang X., Liu H., Wei J.Y., Zhu L., Qian W. (2018). Dual effect of DLBCL-derived EXOs in lymphoma to improve DC vaccine efficacy in vitro while favor tumorgenesis in vivo. J. Exp. Clin. Cancer Res..

[15] Lawrie C.H., Gal S., Dunlop H.M., Pushkaran B., Liggins A.P., Pulford K., Banham A.H., Pezzella F., Boultwood J., Wainscoat J.S. (2008). Detection of elevated levels of tumour-associated microRNAs in serum of patients with diffuse large B-cell lymphoma. Br. J. Haematol..

[16] Matthiesen R., Gameiro P., Henriques A., Bodo C., Moraes M.C.S., Costa-Silva B., Cabeçadas J., da Silva M.G., Beck H.C., Carvalho A.S. (2022). Extracellular Vesicles in Diffuse Large B Cell Lymphoma: Characterization and Diagnostic Potential. Int. J. Mol. Sci..

[17] Feng Y., Zhong M., Zeng S., Wang L., Liu P., Xiao X., Liu Y. (2019). Exosome-derived miRNAs as predictive biomarkers for diffuse large B-cell lymphoma chemotherapy resistance. Epigenomics.

[18] Zare N., Haghjooy Javanmard S., Mehrzad V., Eskandari N., Kefayat A. (2019). Evaluation of exosomal miR-155, let-7g and let-7i levels as a potential noninvasive biomarker among refractory/relapsed patients, responsive patients and patients receiving R-CHOP. Leuk. Lymphoma.

[19] Hurwitz S.N., Nkosi D., Conlon M.M., York S.B., Liu X., Tremblay D.C., Meckes D.G. (2017). CD63 Regulates Epstein-Barr Virus LMP1 Exosomal Packaging, Enhancement of Vesicle Production, and Noncanonical NF-κB Signaling. J. Virol..

[20] Gutzeit C., Nagy N., Gentile M., Lyberg K., Gumz J., Vallhov H., Puga I., Klein E., Gabrielsson S., Cerutti A. (2014). Exosomes Derived from Burkitt’s Lymphoma Cell Lines Induce Proliferation, Differentiation, and Class-Switch Recombination in B Cells. J. Immunol..

[21] Yoon C., Kim J., Park G., Kim S., Kim D., Hur D.Y., Kim B., Kim Y.S. (2015). Delivery of miR-155 to retinal pigment epithelial cells mediated by Burkitt’s lymphoma exosomes. Tumor Biol..

[22] Tang J., Hu P., Zhou S., Zhou T., Li X., Zhang L. (2022). Lymphoma cell-derived extracellular vesicles inhibit autophagy and apoptosis to promote lymphoma cell growth via the microRNA-106a/Beclin1 axis. Cell Cycle.

[23] Ohashi M., Horie K., Hoshikawa Y., Nagata K., Osaki M., Ito H., Sairenji T. (2007). Accumulation of Epstein–Barr virus (EBV) BMRF1 protein EA-D during latent EBV activation of Burkitt’s lymphoma cell line Raji. Microbes Infect..

[24] Damanti C.C., Gaffo E., Lovisa F., Garbin A., Di Battista P., Gallingani I., Tosato A., Pillon M., Carraro E., Mascarin M. (2021). MiR-26a-5p as a Reference to Normalize MicroRNA qRT-PCR Levels in Plasma Exosomes of Pediatric Hematological Malignancies. Cells.

[25] Uccini S., Al-Jadiry M.F., Pepe G., Pasquini A., Alsaadawi A.R., Al-Hadad S.A., Di Napoli A., Tripodo C., Ruco L. (2019). Follicular dendritic cells display microvesicle-associated LMP1 in reactive germinal centers of EBV+ classic Hodgkin lymphoma. Virchows Arch..

[26] Rialland P., Lankar D., Raposo G., Bonnerot C., Hubert P. (2012). BCR-bound antigen is targeted to exosomes in human follicular lymphoma B-cells. Biol. Cell.

[27] Dumontet E., Pangault C., Roulois D., Desoteux M., Léonard S., Marchand T., Latour M., Legoix P., Loew D., Dingli F. (2021). Extracellular vesicles shed by follicular lymphoma B cells promote polarization of the bone marrow stromal cell niche. Blood.

[28] Ferretti E., Tripodo C., Pagnan G., Guarnotta C., Marimpietri D., Corrias M.V., Ribatti D., Zupo S., Fraternali-Orcioni G., Ravetti J.L. (2014). The interleukin (IL)-31/IL-31R axis contributes to tumor growth in human follicular lymphoma. Leukemia.

[29] Caivano A., La Rocca F., Simeon V., Girasole M., Dinarelli S., Laurenzana I., De Stradis A., De Luca L., Trino S., Traficante A. (2016). MicroRNA-155 in serum-derived extracellular vesicles as a potential biomarker for hematologic malignancies—A short report. Cell. Oncol..

[30] Hazan-Halevy I., Rosenblum D., Weinstein S., Bairey O., Raanani P., Peer D. (2015). Cell-specific uptake of mantle cell lymphoma-derived exosomes by malignant and non-malignant B-lymphocytes. Cancer Lett..

[31] Jones R.J., Singh R.K., Shirazi F., Wan J., Wang H., Wang X., Ha M.J., Baljevic M., Kuiatse I., Davis R.E. (2020). Intravenous Immunoglobulin G Suppresses Heat Shock Protein (HSP)-70 Expression and Enhances the Activity of HSP90 and Proteasome Inhibitors. Front. Immunol..

[32] Miguet L., Béchade G., Fornecker L., Zink E., Felden C., Gervais C., Herbrecht R., van Dorsselaer A., Mauvieux L., Sanglier-Cianferani S. (2009). Proteomic Analysis of Malignant B-Cell Derived Microparticles Reveals CD148 as a Potentially Useful Antigenic Biomarker for Mantle Cell Lymphoma Diagnosis. J. Proteome Res..

[33] Star A.T., Hewitt M., Badhwar A., Ding W., Tremblay T.-L., Hill J.J., Willmore W.G., Sandhu J.K., Haqqani A.S. (2025). Comparative Analysis of Plasma Extracellular Vesicle Isolation Methods for Purity Assessment and Biomarker Discovery. Proteomes.

[34] Jia Y., Yu L., Ma T., Xu W., Qian H., Sun Y., Shi H. (2022). Small extracellular vesicles isolation and separation: Current techniques, pending questions and clinical applications. Theranostics.

[35] Upadhya D., Shetty A.K. (2024). MISEV2023 provides an updated and key reference for researchers studying the basic biology and applications of extracellular vesicles. Stem Cells Transl. Med..

[36] Aksamitiene E., Park J., Marjanovic M., Boppart S.A. (2025). Defining Biological Variability, Analytical Precision and Quantitative Biophysiochemical Characterization of Human Urinary Extracellular Vesicles. J. Extracell. Vesicles.

